# Comparison between the effects of progesterone versus corticosteroid local injections in mild and moderate carpal tunnel syndrome: a randomized clinical trial

**DOI:** 10.1186/s12891-015-0752-6

**Published:** 2015-10-26

**Authors:** Mohammad Hassan Bahrami, Shadi Shahraeeni, Seyed Ahmad Raeissadat

**Affiliations:** Physical Medicine and Rehabilitation research center, Shahid Beheshti University of Medical Sciences , Tehran, Iran; Clinical research development center of Shahid Modarres hospital, Shahid Beheshti University of Medical Sciences, Tehran, Iran; Clinical research development center of Shahid Modarres hospital, Shahid Beheshti University of Medical Sciences, Tehran, Iran

**Keywords:** Hydroxyprogesterone, Corticosteroid, Carpal tunnel syndrome

## Abstract

**Background:**

The objective of this study was to compare the short-term effects of progesterone and corticosteroid local injections in the treatment of female patients with carpal tunnel syndrome.

**Methods:**

A randomized clinical trial was used for this study, 60 hands with mild and moderate Carpal Tunnel Syndrome categorized in two groups were used for this study. Patients were treated with a single local injection of triamcinolone acetonide in one group and single local injection of 17-alpha hydroxy progesterone in the other group. Variables such as pain (based on Visual Analogue Scale), symptom severity and functional status (based on Bostone/Levine symptom severity and functional status scale) and nerve conduction study were evaluated before and 10 weeks after the treatments.

**Results:**

Ten weeks after treatment, pain severity and median nerve sensory and motor latencies decreased while patients’ functional status increased meaningfully in both groups. However, there were no meaningful differences between two groups regarding mentioned variables. Pain severity was milder and duration of post-injection pain was shorter in the corticosteroid group. The rates of patient satisfaction were also meaningfully higher in the corticosteroid group.

**Conclusions:**

Both treatments were effective in the short-term management of mild and moderate disease, clinically and electrophysiologically. There were no significant differences in therapeutic effects between two groups.

**Trial registration:**

Current controlled trials IRCT2013101313442N4

## Background

Carpal tunnel syndrome (CTS) is the most common entrapment neuropathy in the upper limb which involves the median nerve at the wrist [1]. Any factor increasing the pressure in this region can cause edema, chronic inflammation and increased soft tissue thickness and compressed median nerve. Patients commonly complain about one or more symptoms of weakness, pain, numbness, paresthesia, especially in the thumb, index and middle fingers which are exacerbated during the night time. this can lead to significant activity restriction, occupational disability and discomfort [2]. Generally treatment is divided into two categories, conservative treatment and surgical release of the median nerve. Surgical treatment is usually used in severe forms of the disease and considering the potential side effects of such treatment. conservative treatments should be used as far as possible especially in mild and moderate forms [3, 4]. These treatments include local injections, medications and physical modalities [5–9]. Existing scientific evidence indicate that in many cases, local corticosteroid injections cause significant relief in patients’ symptoms in mild to moderate CTS, However this effect is not persistent for long term [10, 11]. Progesterone and its derivatives are steroid hormones and apart from their role in reproduction, they also have their neuroprotective effects in the central nervous system according to some studies. Following different damages, Schwann cells start to secrete progesterone which can play a significant role in the acceleration of local inflammation relief and myelin sheath regeneration [12–15]. There are receptors for gonadal steroid hormones such as estrogen and progesterone located on the transverse ligament lining cells and wrist synovial tissue. The number of such receptors increases in patients with CTS. Therefore, it is postulated that local progesterone injection could be effective in conservative treatment of CTS. local corticosteroid injection is the standard treatment known in non-surgical management of CTS. This study was designed to compare the effects of local injections of corticosteroids and progesterone in this disease.

## Methods

Sixty (60) hands of bilateral cases, mild and moderate idiopathic CTS were assessed during single-blinded randomized clinical trial method. Inclusion criteria were as follows: female patients aged between 18 to 60 years referred to physical and rehabilitation medicine clinics of Shohadaye Tajrish and Modarres hospitals with mild and moderate CTS diagnosed by electrodiagnostic studies. Mild CTS was defined clinically as history of nocturnal numbness, paresthesia and electrophysiologically as sensory latency of longer than 3.6 ms with normal motor latency (≤4.2 ms). Moderate CTS was defined clinically as diurnal and nocturnal paresthesia without evidence of atrophy and weakness in thenar muscles and electrophysiologically as sensory latency of longer than 3.6 ms and prolonged motor latency (4.3–6 ms). Exclusion criteria were pregnancy, underlying metabolic diseases such as diabetes mellitus, thyroid diseases, rheumatoid arthritis, history of local corticosteroid injection, severe thenar atrophy, evidence of concomitant neuropathy or radiculopathy and patient’s desire to leave the study.

This research study was approved by the Ethics Committee of ShahidBeheshti University of Medical Sciences, and conducted in accordance with the principles of the Declaration of Helsinki (reference number:400/8770). Also it has was registered in IRCT (Iranian registry of clinical trials).

The methods of progesterone and corticosteroid injection as well as benefits and probable adverse effects were presented by a physiatrist to all patients. The informations listed above were given to the participants in a written form and all participants who signed the consent form were also included in this study.

### Randomization

We used simple random sampling. All hands received a number the by using random numbers table they were allocated to two groups: Progesterone and corticosteroid

Type of study design was parallel groups. Equal allocation between treatment arms was performed. The hands were classified randomly in groups A) progesterone and B)corticosteroid . Random allocation sequence and assignments of hands to interventions were performed by a physiatrist.

### Blinding

Assignments of hands after randomization were placed in sealed envelopes. To ensure blinding, assignments were disclosed to analysis only after the results had been delivered. The patients were unaware of the type of treatment applied. The assessors were unaware of the type of treatment being applied to their hands but the physician was not blinded with respect to treatment.

For all participants, variables of pain (based on VAS), symptom severity and functional status (based on Bostone/Levine symptom severity scale and Bostone/Levine functional status scale, respectively) and sensory and motor distal latencies (using nerve conduction study) were evaluated. Bostone/Levine symptom severity scale (SSS) was used to evaluate the severity of symptoms including pain, paresthesia and weakness during the past 2 weeks. It contained 11 questions with five choices for each one starting from no symptom to very high in severity so that more severe symptoms gained more scores[16]. Bostone/Levine functional status scale (FSS) is used to evaluate the patient’s functional status. It contains eight questions about the patient’s activity for the past 2 weeks and each activity is scored in a scale of five, such that higher scores indicate more inappropriate functional status [16]. Bostone/Levine scales have been validated for Iranian patients [17]. To evaluate pain severity based on visual analogue scale (VAS), patients mark the location on the 10-centimeter line corresponding to the amount of pain they experienced. The point of “no pain” equals to zero and the point of “maximum pain” equals to 10. In electrodiagnostic studies, median sensory and motor distal latencies were evaluated. Median sensory nerve action potential (SNAP) evaluation was done antidromically. The recorder electrode was fixed on the third digit with active-reference distance of 4 cm [18]. Stimulation was applied from the wrist with 14 cm distance from the active electrode. Device sensitivity and sweep speed were set on 20 μv and 2 ms/div, respectively and peak latency was used for measuring the latency. For evaluating the median compound motor action potential (CMAP), recorder electrode was fixed on the abductor pollicis brevis (APB) muscle in such a way that an active electrode was positioned on the most prominent part of the muscle and the reference electrode was positioned on the palmar aspect of the first metacarpophalangeal joint. The median nerve was stimulated supramaximally at a point of 8 cm proximal to the active electrode (measured angularly along the Median nerve course). Device sensitivity and sweep speed were set on 1 mv and 2 ms/div, respectively and onset latency was used for measuring the latency [18]. Hands were divided into two groups (A and B) randomly. Hands in group A received a single local injection of 0.5 ml triamcinolone acetate (40 mg/ml) and 0.5 ml lidocaine (2 %) with a 2-ml syringe using 23-gauge needle. Hands in group B received a single local injection of 0.5 ml 17-alpha hydroxy progesterone (500 mg/2 ml) and 0.5 ml lidocaine (2 %) with the same syringe and needle as group A. Injections were done at 1 cm proximal to wrist crease between the tendons of palmarislongus and flexor carpi radialis with a 45 degree angle. Ten weeks after the injections, patients were visited again and mentioned questionnaires were fulfilled and nerve conduction studies were performed again. For comparing two groups’ means and frequencies, t test and chi square were used, respectively. Finally, the results were analyzed using SPSS version 17.

## Results

Sixty hands with CTS were included in this study (32 hands in the corticosteroid group and 28 hands in the progesterone group). with fifty four hands remaining at the end of the study (30 hands in the corticosteroid group and 24 hands in the progesterone group).

### Pre-intervention findings

Both groups were congruent and similar in mean age, duration and severity of the pain before treatment, sensory and motor latencies and symptom severity based on SSS with no meaningful difference was detected. Patients’ functional status based on FSS was lower in the progesterone group. Demographic variables and nerve conduction characteristics at the beginning of the study in both groups are shown in Table 1.

**Table 1:** Demographic and electrodiagnostic characteristics at the beginning of the study in corticosteroid and progesterone groups

	Variable							
Treatment	*age	#Side ratio RT/total	*Pain (VAS)	*Pain duration (months)	#Severity (Mild/total)	#Dominant hand injection	*DSL	*DML
Corticosteroid	51.7 ± 9.7	62.5 %	5 ± 2.7	16.6 ± 15.2	46.9 %	58 %	4.25 ± 0.59	4.33 ± 0.58
Progesterone	48.2 ± 9.8	53.6 %	4.8 ± 2.4	15.1 ± 13.4	50 %	64.28 %	4 ± 0.33	4.15 ± 0.36
Total	50.07 ± 9.7	55.3 %	5 ± 4.95	15.9 ± 14.3	50 %	60 %	4.12 ± 0.44	4.24 ± 0.47

*chi square test :*P value* >0.05

#t test :*P value* > 0.05

### Ten-week post-intervention changes (Tables 2 and 3)

**Table 2:** Pain severity, symptom and functional status before and 10 weeks after the treatment in both groups

Group	Variable								
	Pain (VAS 1)	Pain (VAS 2)	*Pv within group	Function (FSS 1)	Function (FSS 2)	*Pv within group	Symptom (SSS 1)	Symptom (SSS 2)	*Pv within group
Corticosteroid	5 ± 2.7	2.23 ± 1.30	*P* = 0.0001	1.54 ± 0.50	1.17 ± 0.21	*P* = 0.0001	2.51 ± 0.61	2.07 ± 1.95	*P* = 0.0001
Progesterone	4.8 ± 2.4	2.29 ± 1.75	*P* = 0.0001	1.86 ± 0.56	1.37 ± 0.49	*P* = 0.0001	2.42 ± 0.67	1.70 ± 0.50	*P* = 0.0001

*paired t test

*P* value between groups: *P* value > 0.05(t test)

**Table 3:** Median nerve sensory and motor latencies 10 weeks after the treatment in both groups

Group	Variable					
	distal sensory latency 1	distal sensory latency 2	*Pv within group	motor onset latency 1	motor onset latency 2	*Pv within group
Corticosteroid	4.25 ± 0.59	3.94 ± 0.38	*P* = 0.0001	4.33 ± 0.58	4.04 ± 0.40	*P* = 0.003
Progesterone	4 ± 0.33	3.85 ± 0.42	*P* = 0.009	4.15 ± 0.36	4.02 ± 0.49	*P* = 0.014

*paired t test

*P* value between groups: *P* value > 0.05(t test)

Pain (based on VAS), symptom severity (based on Boston SSS), functional status (based on Boston FSS) and electrodiagnostic variables were improved upon in both groups significantly. However, there was no meaningful difference between the two groups when compared.

Patients were evaluated for disease severity based on electrodiagnostic studies before and 10 weeks after treatment. The NCS became normal in 20 % of patients in the corticosteroid group and 25 % of patients in the progesterone group (totally 22.2 %) at the end of the study (Table 4). There was no statistical difference between the two groups in the disease severity changes.

**Table 4:** Disease severity based on electrodiagnostic findings before and 10 weeks after the treatment in both groups

Group	Variable					
	Severity1(%)		Severity2(%)			
	Mild	Moderate	Normal	Mild	Moderate	* p.v within group
Progesterone	53.6 %	46.4 %	25 %	58.3 %	16.7 %	0.32
Corticosteroid	46.9 %	53.1 %	20 %	60 %	20 %	0.57
Total	50 %	50 %	22.2 %	59.3 %	18.5 %	

*chi square test

*P* value between groups: *P* value > 0.05 (chi square test)

There was some degree of post-injection pain in most patients in the progesterone group. Post-injection pain severity (based on VAS) and duration in the progesterone group was more than the corticosteroid group (*p* = 0.001). Patient satisfaction injection was meaningfully greater in the corticosteroid group 10 weeks after the injection was assessed (*p* = 0.005). There was no meaningful relationship between patient satisfaction and post-injection pain severity and duration and also between symptom severity and functional status improvement and sensory and motor latency changes.

## Discussion

Multiple non-surgical treatments have been proposed for the treatment of CTS including oral medications such as nonsteroidal anti-inflammatory drugs (NSAIDs), oral corticosteroids and supplemental vitamin B6 and physical modalities such as ultrasound, low-power laser, polychromatic polarized light (Bioptron), exercise therapy, nerve mobilization, acupuncture, splinting and local steroid injection. New treatments under investigation are local insulin and progesterone injections. Hydroxy progesterone is a natural progesterone hormone metabolite which have been shown in different studies to have neuroprotective effects in central and peripheral nervous systems [12–14, 19]. It has been shown that Schwann cells start to secrete these steroids in response to peripheral nervous system injuries which leads to Schwann cell proliferation and increased myelin synthesis [20–22]. However there are limited studies about the effects of progesterone and its derivatives in the treatment of entrapment neuropathies these include comparison between local injection of triamcinolone with 17-hydroxy progesterone in mild carpal tunnel syndrome. In this study patients were divided into two groups with 8 participants in each one. Patients were randomly allocated to one of two groups: (I) single corticosteroid (triamcinolone acetonide, 20 mg/0.5 ml,Triamvigi, Fisiopharma), or (II) single synthetic derivative of PROG (17HPC, 85 mg/0.5 ml, Lentogest, A.M.S.A.) Patients’ symptoms based on VAS pain scale, Boston questionnaires for SSS and FSS, and electrodiagnostic findings were all evaluated 1 and 6 months after the injections. Pain severity in both groups decreased after 1 month and there was no meaningful difference between two groups. However, this effect remained only in the progesterone group after 6 months. Improved functional scales were only seen in progesterone group while improved electrodiagnostic findings were only demonstrated in corticosteroid group, implicating the long term effect of local progesterone injection on clinical improvement of CTS compared to corticosteroid [23]. In our study mild and moderate CTS were evaluated but in previous study only subjects with mild CTS were selected and dosage of injected drug was different from. In our study, in 10-weeks follow up, variables of pain, symptom severity (SSS), functional status (FSS) and electrodiagnostic findings were improved significantly in both groups with no meaningful difference between the two groups. No statistical difference was shown in severity change based on electrodiagnostic criteria in any of the groups.

Multiple studies have been performed about local corticosteroid injection in CTS treatment. In a study camed out in 2011, local injection of triamcinolone was compared with procaine. Pain was improved more in corticosteroid group after 2 months. However, after 6 months of follow up no meaningful difference was seen[24]. In another study, 69 patients were divided into two groups. In one group, local injection of triamcinolone was administered while in the other group, local injection of saline (as placebo) was administered and patients’ symptoms were evaluated using SSS and FSS. In the short-term follow up, significant improvement was seen in the first group after 1 month. The main mechanism of action of steroid in CTS treatment is through decrease in flexor retinaculum inflammation and swelling and the resulting decrease in median nerve pressure [25]. In a review article published in Cochrane (2007), 12 studies including 671 patients were evaluated and it was shown that local corticosteroid injection leads to obvious clinical improvement in patients in 1-month follow up compared to placebo. However, no significant improvement was detected after 1 month and it was recommended that more studies were needed to be performed to determine the long-term effects of steroid injections in mild and moderate CTS [11]. In our study, all of the variables of pain, symptom severity, functional status and electrodiagnostic findings significantly improved after 10 weeks of follow up. According to some studies, the probable mechanism of action of progesterone on patients’ pain and symptoms may be due to decrease in pain and paresthesia generating impulses through regulating voltage-gated sodium channels, the amount of calcium flow and Na/K ATPase pump activity. On the other hand, progesterone has influence on opioid receptors located on non-myelinated sensory nerves. This causes an increase in the peripheral tissue inflammatory processes thereby inhibiting the increase in P substance; therefore leading to pain decrease. Progesterone’s effect on improved myelin synthesis, is the main primary pathology in CTS. This is the probable explanation for improved electrodiagnostic findings in such patients.

## Conclusions

According to this study, both corticosteroid and progesterone are effective in short-term improvement of electrodiagnostic and clinical findings of mild and moderate CTS. However, there is no statistical difference between the two groups. Due to the probable patients drop out in long term follow up, 10 week follow up was considered for this study. The lack of control group and rather small sample size are among other study limitations. In order to gain more definite results, performing a study with larger sample size and longer follow up period with including the control group with no therapeutic intervention and also objective evaluation of patients’ functional status such as grip power assessment seem necessary.
